# Supplementary material for: Detailed molecular and epigenetic characterization of the pig IPEC-J2 and chicken SL-29 cell lines
Source: iScience. 2023 Feb 20;26(3):106252. doi: 10.1016/j.isci.2023.106252 (PMC10018572; doi:10.1016/j.isci.2023.106252)
Supplement: Data S2. Complete homer output for identified motifs in Chicken SL-29, related to Tables 5 and 6 — Homer motif analysis results for histone modifications H3K4me1, H3K4me3, H3K27ac, enhancers, and ATAC-seq of chicken SL-29 cell line. Parameters for possible false positives is as mentioned earlier for S5. [file mmc3.zip › Data_S2/S6/Chicken_SL_29/peak_files_CTCF/homerResults/motif11.similar.html]

motif11

## Information for motif11

T
A
G
C
A
T
C
G
A
T
C
G
A
T
G
C
A
T
C
G
A
C
G
T
A
C
G
T
A
G
C
T
A
G
C
T
A
G
C
T
  
Reverse Opposite:  

T
C
G
A
T
C
G
A
T
C
G
A
T
G
C
A
T
G
C
A
T
A
G
C
T
A
C
G
T
A
G
C
T
A
G
C
A
T
C
G
  

|  |  |
| --- | --- |
| p-value: | 1e-68 |
| log p-value: | -1.578e+02 |
| Information Content per bp: | 1.445 |
| Number of Target Sequences with motif | 13189.0 |
| Percentage of Target Sequences with motif | 44.74% |
| Number of Background Sequences with motif | 11551.2 |
| Percentage of Background Sequences with motif | 39.71% |
| Average Position of motif in Targets | 148.6 +/- 84.8bp |
| Average Position of motif in Background | 149.1 +/- 95.8bp |
| Strand Bias (log2 ratio + to - strand density) | 0.0 |
| Multiplicity (# of sites on avg that occur together) | 1.40 |
| Motif File: | file (matrix) reverse opposite |

### Similar de novo motifs found

|  |  |  |  |  |  |  |  |
| --- | --- | --- | --- | --- | --- | --- | --- |
| Rank | Match Score | Redundant Motif | P-value | log P-value | % of Targets | % of Background | Motif file |
| 1 | 0.737 | C G T A G T C A T C G A T A G C G T A C G T A C T G A C C A T G | 1e-63 | -146.805585 | 38.09% | 33.40% | motif file (matrix) |
